# Intersections of Autism and Sexual and Gender Diversity Among Young People in New Zealand

**DOI:** 10.1177/13623613261460933

**Published:** 2026-07-08

**Authors:** Brodie Fraser, Sujata Saha, Mary Buchanan, Sheree Gibb, Ruth Monk, Laurie K. McLay, Phoebe Jean Campbell, Jordan Rogers, Nicholas James Bowden

**Affiliations:** 1 University of Otago Wellington, New Zealand; 2 University of Otago, Dunedin, New Zealand; 3 University of Canterbury, Christchurch, New Zealand

**Keywords:** Autism, sexual and gender diversity, children, youth, New Zealand, big data

## Abstract

International research suggests there is a common intersection between Autism and sexual and gender diversity. However, there is limited data estimating the whole-of-population prevalence of this association, both abroad and in Aotearoa New Zealand (NZ). Utilising NZ’s Integrated Data Infrastructure, a research database containing linked administrative and survey data, we conducted a nationwide cohort study of those in the age range 5–24 years for whom Census 2023 records existed. From a study population of 1,266,453 young people, 1.5% were identified as Autistic. After adjustment for demographic measures, we found significantly higher rates of Autism among sexually- and gender-diverse young people than among their heterosexual and cisgender peers. This included for those identifying with a gender outside of the male/female binary (3.9%) compared to both males (2.3%; 1.7 times as high) and females (0.7%; 5.8 times as high), transgender people (4.0%) compared to cisgender people (1.5%; 2.9 times as high), and non-heterosexuals (2.2%) compared to heterosexuals (1.4%; 1.8 times as high).

**Lay Abstract:**

International research suggests there is a common overlap between Autism and sexual and gender diversity (such as people who are gay, lesbian, transgender, etc). However, there is little data about this association on a whole-of-population scale. We conducted a nationwide study of young people aged 5–24 years to explore this crossover. From a study population of 1,266,453 young people, 1.5% were identified as Autistic. Looking at sexual- and gender-diversity data, we found there were significantly higher proportion of people who were Autistic among sexually and gender-diverse young people compared to their heterosexual and cisgender peers.

## Introduction

Evidence suggests there is a relatively common intersection of Autism and sexual and gender diversity (Adams et al., 2024; Dewinter et al., 2017; George & Stokes, 2018; McQuaid et al., 2023; Strauss et al., 2021; Tollit et al., 2024; Warrier et al., 2020). Existing studies have typically been limited to small-scale surveys or clinical samples, restricting generalisability. To our knowledge, no studies have utilised whole-of-population data to examine this association. Following the inclusion of questions capturing sexual and gender diversity in New Zealand’s (NZ’s) 2023 Census, we can explore what these statistics look like on a whole-of-population scale for the first time. This short report focuses specifically on young people, where the administrative data on Autism is most reliable, looking at the rates of Autism among sexuality- and gender-diverse (SGD) young people.

NZ Census 2023 data shows SGD people comprise 4.9% (172,383 people) of the adult population (Statistics New Zealand, 2025). The SGD population were more likely to be younger, live in urban areas, have lower incomes, and experience homelessness and other housing difficulties (Statistics New Zealand, 2025). SGD people are more likely to report a disability of some kind; this is most pronounced for young people (Statistics New Zealand, 2025). Other research also shows high rates of disability (Fenaughty et al., 2022; Fleming et al., 2021; Fraser et al., 2022) and neurodivergence (Yee et al., 2025), including Autism, among SGD New Zealanders.

Providing robust statistics on the intersection between Autism and SGD young people is important for several reasons. Namely, evidence shows people with these intersecting identities face poorer mental and physical wellbeing outcomes, such as lower reported quality of life, higher rates of self-harm and suicidality, and worse self-reported physical health, and higher unmet healthcare needs than those who do not sit at this intersection (Adams et al., 2024; McQuaid et al., 2023; Strauss et al., 2021; Tollit et al., 2024). Other research explores people’s experiences of dual identities and minority stressors (Hillier et al., 2020), intersectional and compounding health disparities (Hall et al., 2020), and the importance of ensuring an Autism diagnosis in and of itself does not serve as an exclusion criterion for transgender (Bo et al., 2024). There is thus a need for greater awareness of these intersections to ensure the development of strategies for equitable outcomes. The objective of this study was to utilise linked population-level data from the 2023 NZ Census and administrative health records to explore rates of Autism among SGD children and young people compared to the wider population.

## Methods

### Study Design

This was a nationwide cohort study of participants aged 5–24 years who completed the 2023 NZ Census. These data were accessed using the Integrated Data Infrastructure (IDI), a secure research database managed by StatsNZ that links anonymised administrative and survey data across government agencies to enable population-level analysis of social, health, and economic outcomes (Milne et al., 2019).

### Autism

Autism was identified using an established IDI-based case-identification method (Bowden et al., 2020) drawing on diagnosis information from three health datasets: hospitalisation (the National Minimum Dataset [NMDS]), specialist mental health (the Programme for the Integration of Mental Health Data [PRIMHD]), and disability support services data (Socrates). Individuals were identified as Autistic if a lifetime diagnosis code for Autism was present in any of the three datasets (see Appendix Table 1 for details).

### Gender and Sexuality Indicators

The 2023 Census asked about gender (male, female, another gender), sex at birth (male, female), and sexuality (see Appendix Table 2 for the Census questions). Utilising responses to the sex at birth and gender questions, StatsNZ generated a categorical transgender status variable which included ‘cisgender male’, ‘cisgender female’, ‘transgender male’, ‘transgender female’, and ‘transgender other’ (see Appendix Table 3 for details of how transgender categories were derived). For this study, we collapsed these responses into a binary (transgender/cisgender) variable, with only this binary variable used in analysis. StatsNZ categorised responses to the sexuality question as ‘heterosexual’, ‘homosexual’, ‘bisexual’, or ‘sexual identity not elsewhere classified’, and the question was restricted to those aged 15 years or older; unlike the gender question, which was asked of everyone. These were also collapsed into a binary (heterosexual/non-heterosexual) variable. Both the specific responses to the sexuality question and the binary variable were used in the analyses. There were high levels of missing data for these variables including 11.8% for gender, 18.2% for transgender status, and 27.2% for sexuality. Some missing data were imputed by StatsNZ (Statistics New Zealand, 2024); however, for this study, only ‘self-reported’ 2023 Census data were used. For those aged under 15 years, parents/caregivers are responsible for ensuring forms are completed, and therefore, may have completed on the child’s behalf.

### Sociodemographic Variables

Age (5–9, 10–14, 15–19, 20–24 years), ethnicity (Asian; European; Māori; Middle Eastern, Latin American African [MELAA]; Other, Pacific), deprivation level, and urban/rural profile of residence were all derived from 2023 Census data. Age was measured in years as at Census night; we chose to begin the analysis at age five, as this is consistent with the literature regarding the average age at which gender diversity is first expressed (Holt et al., 2016; Tollit et al., 2023). Ethnicity was measured using Level 1 Groupings using the total response format meaning individuals could identify with multiple ethnic groups. Deprivation level reflects area-level socioeconomic disadvantage and was derived from the New Zealand Index of Deprivation (NZDep) 2023, a composite measure based on census variables including income, employment, housing, education, and access to transport and communication. Meshblocks of usual residence were linked to NZDep scores, which were collapsed into quintiles, where one represents areas of the least deprivation, and five indicates areas of the highest deprivation. Similarly, meshblocks were linked to the 2023 StatsNZ urban/rural classification and measured as a binary urban/rural indicator.

### Statistical Analysis

All analyses were undertaken using Stata/MP 19.5 within the IDI. Initially, a descriptive summary of the study population was presented, stratified by Autism status. This was followed by observed and standardised rates of Autism by gender, transgender status, and sexuality. Standardised Autism rates were estimated using logistic regression models that adjusted for age, ethnicity, deprivation, and urban/rural variables. Standardisation was then applied using the margins command to generate adjusted prevalence estimates for each group, holding the covariate distribution constant across the population.

### Community Involvement

Our research recognises the importance of engaging meaningfully with both the Autistic and SGD communities, with a strong emphasis on collaboration and co-production of knowledge. The authorship team includes multiple contributors from the SGD community, from the Autistic community, and individuals who identify as both Autistic and SGD, ensuring these perspectives are directly represented throughout the research process.

## Results

The study population included 1,266,453 young people aged 5–24 years in the 2023 NZ Census. Among those, 18,699 (1.47%) were identified as Autistic. Table 1 presents the demographic characteristics of the study population by Autism status. Compared with the non-autistic population, Autistic individuals were slightly under-represented in the younger (5–9 years) and older (20–24 years) age groups. They were over-represented among Europeans, while Asian and Pacific peoples were under-represented. Distributions by deprivation quintile were similar across both groups, as was the urban–rural profile of residence.

**Table 1.** Demographic Characteristics of the Study Participants (Aged 5–24 Years).

	Total		Non-autistic		Autistic	
	n	%	n	%	n	%
Total	1,266,453		1,247,754		18,699	
Age						
5–9	313,371	24.7	309,324	24.8	4,047	21.6
10–14	334,473	26.4	328,932	26.4	5,541	29.6
15–19	311,703	24.6	306,423	24.6	5,280	28.2
20–24	306,909	24.2	303,081	24.3	3,828	20.5
Ethnicity^a^						
Asian	223,365	17.6	220,788	17.7	2,577	13.8
European	818,259	64.6	804,246	64.5	14,013	74.9
Māori	328,662	26.0	324,027	26.0	4,635	24.8
MELAA	26,817	2.1	26,460	2.1	357	1.9
Other	11,760	0.9	11,511	0.9	249	1.3
Pacific	176,097	13.9	174,159	14.0	1,938	10.4
Deprivation quintiles^b^						
1 (least deprived)	235,593	18.6	232,407	18.6	3,186	17.0
2	232,122	18.3	228,657	18.3	3,465	18.5
3	236,403	18.7	232,644	18.6	3,759	20.1
4	251,475	19.9	247,638	19.8	3,837	20.5
5 (most deprived)	310,746	24.5	306,297	24.5	4,449	23.8
Urban/rural^c^						
Urban	1,082,115	85.4	1,065,822	85.4	16,293	87.1
Rural	184,206	14.5	181,800	14.6	2,406	12.9

MELAA = Middle Eastern, Latin American, African.

a Percentages will sum to over 100% due to the possibility of identifying with multiple ethnic groups in NZ.

b Missing values for 114 individuals.

c Missing values for 132 individuals.

Table 2 presents observed and standardised Autism rates by gender, transgender, and sexuality measures. Observed data show 4.44% of those with a gender outside the male/female binary were Autistic, compared to 2.25% of males and 0.68% of females. Similarly, 4.46% of the transgender population were Autistic. In terms of sexual orientation, 1.37% of heterosexual young people were Autistic, and 2.42% of non-heterosexuals were Autistic. The proportion of those who were Autistic is noticeably higher among those in the ‘sexual identity not elsewhere categorised’ group, which includes, for example, those who prefer the label queer to describe their sexuality. Standardised rates present similar trends, with Autism rates significantly higher among those identifying with a gender outside of the male/female binary (3.93%) compared to both male (2.25%; 1.7 times as high) and female (0.68%; 5.8 times as high) rates. The rate of Autism was also significantly higher among transgender people (4.00%) than among cisgender people (1.52%; 2.9 times as high), and higher among non-heterosexuals (2.22%) than among heterosexuals (1.38%; 1.8 times as high). For more details of the distribution of census responses, including missing data, see Appendix Table 4. Observed rates of Autism by age are reported in Appendix Tables 5–7.

**Table 2.** Observed and Standardised Population Rates (%) of Autism by Gender, Transgender, and Sexuality Among Those Who Completed the 2023 NZ Census.

	n	Autism rate (%)	Standardised^a^ Autism rate (%)	95% CI for standardised rate
Gender				
Male	12,810	2.25	2.25	2.22–2.29
Female	3,654	0.68	0.68	0.66–0.70
Other	387	4.44	3.93	3.54–4.32
Transgender				
Yes	585	4.46	4.00	3.68–4.32
No	15,576	1.52	1.52	1.50–1.55
Sexuality				
Heterosexual	5,520	1.37	1.38	1.34–1.42
Non-heterosexual (total)	1,110	2.42	2.22	2.09–2.35
Homosexual	264	2.98	2.76	2.43–3.09
Bisexual	552	1.86	1.71	1.56–1.85
Sexual identity not elsewhere classified	294	4.07	3.64	3.23–4.05

a Standardised by age, ethnicity, deprivation, and urban/rural.

## Discussion and Conclusion

This short report shows higher proportions of SGD young people are Autistic, compared to non-SGD young people. This holds true after rates have been standardised by age, ethnicity, geographic area deprivation, and urban or rural residence. These findings align with national and international research showing a common intersection between Autism and sexual and gender diversity. The uniqueness of this report lies in the use of whole-of-population data, which provides robust evidence confirming such intersections are common across the entire population of young people in NZ. While we observed higher rates of Autism among SGD young people, this association is not universal, and most SGD individuals are not Autistic; these findings should not be interpreted as explanatory of broader health disparities, which are shaped by a range of social and structural factors.

Our findings show that among all groups we looked at, transgender people had the highest rates of Autism diagnosis. Autistic people often report conceptualising gender differently from non-autistic peers, with less adherence and pressure to conform to social conditioning/norms (Kristensen & Broome, 2015; Strang et al., 2018; Walsh et al., 2018). This can lead to earlier and/or more open recognition of gender as a social construct, exploration of gender, and expression of gender that challenges binary gender expectations (Kristensen & Broome, 2015; Strang et al., 2018; Walsh et al., 2018). However, research also shows for transgender young people, their access to gender-affirming healthcare can be restricted by paternalistic norms which perpetuate the misconception that because a young person is Autistic, they cannot be trusted to know their own gender (Adams et al., 2024; Turban & van Schalkwyk, 2018). The intersection of these two identities should not preclude people from accessing vital healthcare (Coleman et al., 2022).

Few NZ studies have examined the intersections of Autism and SGD identities. A recent exception comes from a master’s thesis focused on the mental health of Autistic transgender youth, using data from the Counting Ourselves survey. This research found a high rate of poor mental health among trans people who were Autistic, and additional qualitative data revealed findings around identity journeys, finding acceptance, societal stigma, and inner experiences (Jones, 2024). Autistic and trans identities were intimately linked for these young people, and they faced high levels of intersecting stigma, which contributed to poor mental wellbeing (Jones, 2024). Taken together with our findings, there is a need to explore, understand, and consider the unique needs of Autistic SGD young people to inform policy, and for health and education delivery, to ensure equity of outcomes for these young people.

### Limitations

This method for identifying formally diagnosed Autistic people in the population remains unvalidated, so the extent to which it identified false positives and false negatives is unknown. Moreover, it relies on health service use data, which does not include diagnosis information from primary care or outpatient settings. Thus, it likely undercounts the true population of diagnosed Autistic people in NZ. It also does not include those who have not been able to access a formal diagnosis of any kind. However, this is an established method used extensively in the literature (Bowden et al., 2022; McLay et al., 2025; Mujoo et al., 2023; Schluter et al., 2025; Vu et al., 2024). In addition, the use of Diagnostic and Statistical Manual of Mental Disorders, 4th edition (*DSM*-IV)-era diagnostic codes may result in the inclusion of conditions that are not part of the current *DSM*-5 classification of Autism spectrum disorder. In particular, Rett syndrome, previously classified within Pervasive Developmental Disorder (PDD) categories but no longer considered part of Autism, may have been captured, introducing a potential source of misclassification.

Our analyses relied on 2023 Census data to identify key variables, including those relating to gender and sexuality. One limitation of this is that parents are responsible for ensuring census forms for under-15s are filled out; they are not required to fill out the form on their child’s behalf, but many do. Some children will have filled out their forms themselves, and some would have had parents do it for them. Thus, there is likely to be an undercount of the SGD population in cases such as those where a young person is not yet ‘out’ to their family. In addition, we chose to restrict this analysis to only ‘self-report’ responses in the 2023 Census to ensure optimal reliability of these variables, that is, we did not include those that were imputed by StatsNZ. However, by limiting to self-reported data, this increases the extent of missing data and may limit the generalisability of these findings to the whole NZ population. The 2023 Census was the first time sexuality and ‘another gender’ options had been included in a NZ Census. The lack of a historical time series for these data, and an absence of other sources of whole-population data for validation, means we know little about the accuracy of the data and how well they represent the SGD community in NZ.

The findings of this report show there is a need for future research on the intersections of Autism and SGD identities. This report serves as a starting point to show the prevalence of intersecting Autistic and SGD identities. Further research looking at the demographics of those who sit at this intersection is needed, as well as other research that focuses on, for example, the healthcare and educational needs and outcomes of this group. Our findings show transgender people and those with less-common sexual orientations (i.e. those in the ‘sexuality not elsewhere categorised’ group) were more likely to be Autistic than any other group we looked at. Future research that investigates the experiences of these groups specifically is encouraged to ensure equity of outcomes.

## Appendix 1

**Table 1.** Diagnostic Codes for Identifying Autism.

Dataset	Code type	Code	Code description
NMDS, PRIMHD	ICD-10-AM	F84.0	Autistic disorder
NMDS, PRIMHD	ICD-10-AM	F84.1	Atypical Autism
NMDS, PRIMHD	ICD-10-AM	F84.3	Other childhood disintegrative disorder
NMDS, PRIMHD	ICD-10-AM	F84.5	Asperger’s syndrome
NMDS, PRIMHD	ICD-10-AM	F84.8	Other pervasive developmental disorders
NMDS, PRIMHD	ICD-10-AM	F84.9	Pervasive developmental disorder, unspecified
PRIMHD	*DSM*-IV	299.00	Autistic disorder
PRIMHD	*DSM*-IV	299.10	Other childhood disintegrative disorder
PRIMHD	*DSM*-IV	299.80	Asperger’s disorder/pervasive development disorder not otherwise specified
Socrates	Assigned Diagnosis	1206	Asperger’s syndrome
Socrates	Assigned Diagnosis	1207	Other ASD
Socrates	Assigned Diagnosis	1211	Autism spectrum disorder

*Note. NMDS, National Minimum Dataset*; PRIMHD, Programme for the Integration of Mental Health Data; ICD-10-AM, International Statistical Classification of Diseases and Related Health Problems, Tenth Revision, Australian Modification; *DSM*-IV, Diagnostic and Statistical Manual of Mental Disorders, 4th edition; ASD, autism spectrum disorder.

**Table 2.** 2023 Census Questions.

Question	Response options
What is your gender?	‘male’, ‘female’, and ‘another gender’
What was your sex at birth?	‘male’ and ‘female’
Which of the following best describes how you think of yourself?	‘heterosexual/straight’, ‘gay or lesbian’, ‘bisexual’, ‘another identity, please state’

**Table 3.** Transgender Variable Derivation.

Transgender group	Sex at birth question response	Gender question response
Cisgender male	Male	Male
Cisgender female	Female	Female
Transgender male	Female	Male
Transgender female	Male	Female
Transgender other	Male or Female	Another gender

**Table 4.** Observed 2023 NZ Census Responses Among the Study Participants (Aged 5–24 Years).

	Total		Non-autistic		Autistic	
	n	%	n	%	n	%
Total	1,266,453		1,247,754		18,699	
Gender						
Male	568,677	44.9	555,864	44.5	12,813	68.5
Female	539,970	42.6	536,316	43.0	3,654	19.5
Other	8,718	0.7	8,331	0.7	387	2.1
Missing	149,088	11.8	147,243	11.8	1,845	9.9
Transgender status						
Yes	13,107	1.0	12,522	1.0	585	3.1
No	1,023,363	80.8	1,007,787	80.8	15,576	83.3
Missing	229,983	18.2	227,445	18.2	2,538	13.6
Specific transgender categories						
Cisgender male	523,950	50.6	511,752	50.2	12,198	75.5
Cisgender female	499,413	48.2	496,035	48.6	3,378	20.9
Transgender male	2,565	0.2	2,463	0.2	102	0.6
Transgender female	2,409	0.2	2,304	0.2	105	0.6
Transgender other	8,133	0.8	7,758	0.8	375	2.3
Sexuality (15–24 years, *N* = 618,612)						
Heterosexual	404,298	65.4	398,778	65.4	5,520	60.6
Non-heterosexual	45,816	7.4	44,706	7.3	1,110	12.2
Homosexual	8,850	1.4	8,586	1.4	264	2.9
Bisexual	29,745	4.8	29,193	4.8	552	6.1
Sexual identity not elsewhere classified	7,221	1.2	6,927	1.1	294	3.2
Missing	168,495	27.2	166,017	27.2	2,478	27.2

**Table 5.** Observed Rates of Autism by Gender and Age Among 2023 NZ Census Respondents.

			n	%
Male	Total		12,810	2.3
	Age (years)	5–9	2,727	1.9
		10–14	3,918	2.6
		15–19	3,573	2.5
		20–24	2,592	1.9
Female	Total		3,654	0.7
	Age (years)	5–9	765	0.6
		10–14	1,032	0.7
		15–19	1,053	0.8
		20–24	804	0.6
Other	Total		387	4.4
	Age (years)	5–9	12	2.6
		10–14	63	4.7
		15–19	198	5.9
		20–24	114	3.2

**Table 6.** Observed Rates of Autism by Transgender Status and by Age Among 2023 NZ Census Respondents.

			n	%
Transgender	Total		582	4.4
	Age (years)	5–9	18	1.7
		10–14	96	4.3
		15–19	285	5.7
		20–24	183	3.8
Non-transgender	Total		15,576	1.5
	Age (years)	5–9	3,300	1.3
		10–14	4,695	1.7
		15–19	4,371	1.8
		20–24	3,210	1.3
Cisgender male	Total		12,195	2.3
	Age (years)	5–9	2,583	2.0
		10–14	3,732	2.6
		15–19	3,405	2.6
		20–24	2,475	2.1
Cisgender female	Total		3,384	0.7
	Age (years)	5–9	717	0.6
		10–14	963	0.7
		15–19	966	0.8
		20–24	738	0.6
Transgender male	Total		102	4.0
	Age (years)	5–9	S^a^	S
		10–14	S	S
		15–19	57	5.1
		20–24	27	3.8
Transgender female	Total		108	4.5
	Age (years)	5–9	9	2.2
		10–14	15	3.0
		15–19	39	5.1
		20–24	45	6.1
Transgender other	Total		375	4.6
	Age (years)	5–9	12	2.9
		10–14	60	4.9
		15–19	192	6.1
		20–24	111	3.3

a S: Suppressed by StatsNZ due to low counts.

**Table 7.** Observed Rates of Autism by Sexuality and Age Among 2023 NZ Census Respondents.

			n	%
Heterosexual	Total		5,517	1.4
	Age (years)	15–19	3,213	1.6
		20–24	2,304	1.2
Non-heterosexual	Total		1,116	2.4
	Age (years)	15–19	594	3.1
		20–24	522	2.0
Homosexual	Total		264	3.0
	Age (years)	15–19	144	3.8
		20–24	120	2.4
Bisexual	Total		552	1.9
	Age (years)	15–19	279	2.3
		20–24	273	1.6
Sexual identity not elsewhere classified	Total		294	4.1
	Age (years)	15–19	168	5.3
		20–24	126	3.1
